# Acute Pancreatitis-Induced Thrombosis of Celiac Artery: An Unusual Complication of Acute Pancreatitis

**DOI:** 10.7759/cureus.46249

**Published:** 2023-09-30

**Authors:** Ali Tariq Alvi, Luis E Santiago, Marvin Lopez-Medal

**Affiliations:** 1 Internal Medicine, HCA Florida Westside Hospital, Plantation, USA; 2 Internal Medicine, HCA Florida Northwest Hospital, Margate, USA

**Keywords:** acute arterial thrombosis, celiac trunk, acute abdomen, complicated acute pancreatitis, acute gallstone pancreatitis

## Abstract

Acute pancreatitis can lead to both local and systemic complications, including pseudocysts, biliary obstruction, duodenal obstruction, sepsis, necrosis, vascular complications, and multiorgan failure. Vascular complications following acute pancreatitis are associated with a high risk of morbidity and mortality due to their thrombotic and hemorrhagic effects. When thrombosis is present, it usually involves the splanchnic venous system, but it is rarely seen in the arterial system. Celiac artery thrombosis is rare with only a few cases reported in the literature. In this case, we present a 65-year-old Hispanic female who presented to the emergency department with abdominal pain and nausea, with computed tomography angiography (CTA) of the abdomen revealing acute pancreatitis with thrombosis of the celiac artery, which was managed with anticoagulation.

## Introduction

According to one estimate, there are more than 210,000 hospital admissions per year for acute pancreatitis and more than 56,000 admissions per year for chronic pancreatitis in the United States [1]. Acute pancreatitis is an acute inflammation of the pancreas, mostly associated with a history of gallstones or excessive alcohol use and characterized by local tissue injury causing systemic inflammatory response [2]. Gallstones are the most common cause and account for 40-70% of cases of acute pancreatitis [3], followed by alcohol use, which accounts for 25-35% of cases in the United States [4]. Other less common etiologies include hypertriglyceridemia, trauma from endoscopic retrograde cholangiopancreatography, medication-induced causes, hypercalcemia, abdominal trauma, various infections, or genetic causes [5,6]. Patients commonly present with sudden onset of abdominal pain located generally in the epigastrium, with radiation to the back in almost half of the cases, associated with nausea and vomiting [7]. The diagnosis of acute pancreatitis requires two of the following three features: serum lipase or amylase greater than three times the upper limit of the normal range, abdominal pain clinically consistent with acute pancreatitis, and computed tomography scan showing characteristic findings of acute pancreatitis [7,8].

Acute pancreatitis can lead to both local and systemic complications depending on the severity. Based on the Revised Atlanta Classification of Acute Pancreatitis, the disease can be classified into mild, moderately severe, or severe. The most common type is mild acute pancreatitis, which resolves in the first week and is associated with no local or systemic complications or organ failure. Moderately severe disease is associated with local or systemic complications or the presence of transient organ failure that resolves within 48 hours. Severe acute pancreatitis involves single or multiple organ failure that is persistent for greater than 48 hours [8]. Local complications include pancreatic and peripancreatic necrosis, peripancreatic fluid collection, pseudocyst, vascular complications, and walled-off necrosis [8,9]. Vascular complications commonly involve the formation of pseudoaneurysms, thrombosis, and hemorrhages. Thrombosis mostly involves portosplenomesenteric venous system and is rarely seen with the arterial system [9].

## Case presentation

**Figure 1:**
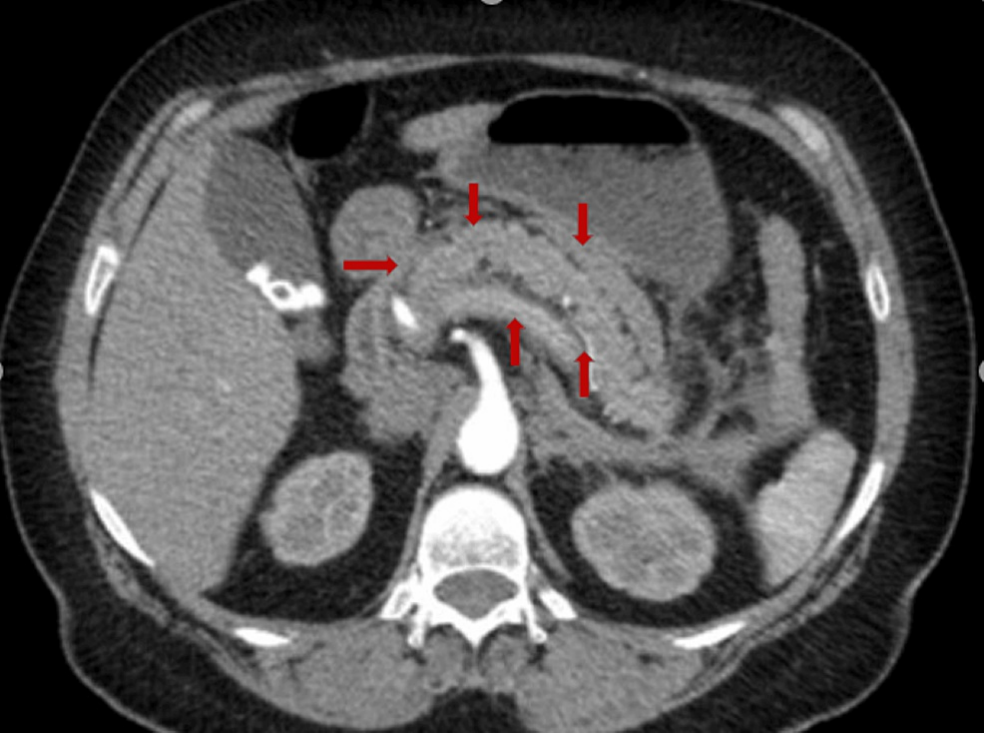
Computed tomography angiography (CTA) of the abdomen showing diffuse pancreatic enlargement with edema (red arrows).

**Figure 2:**
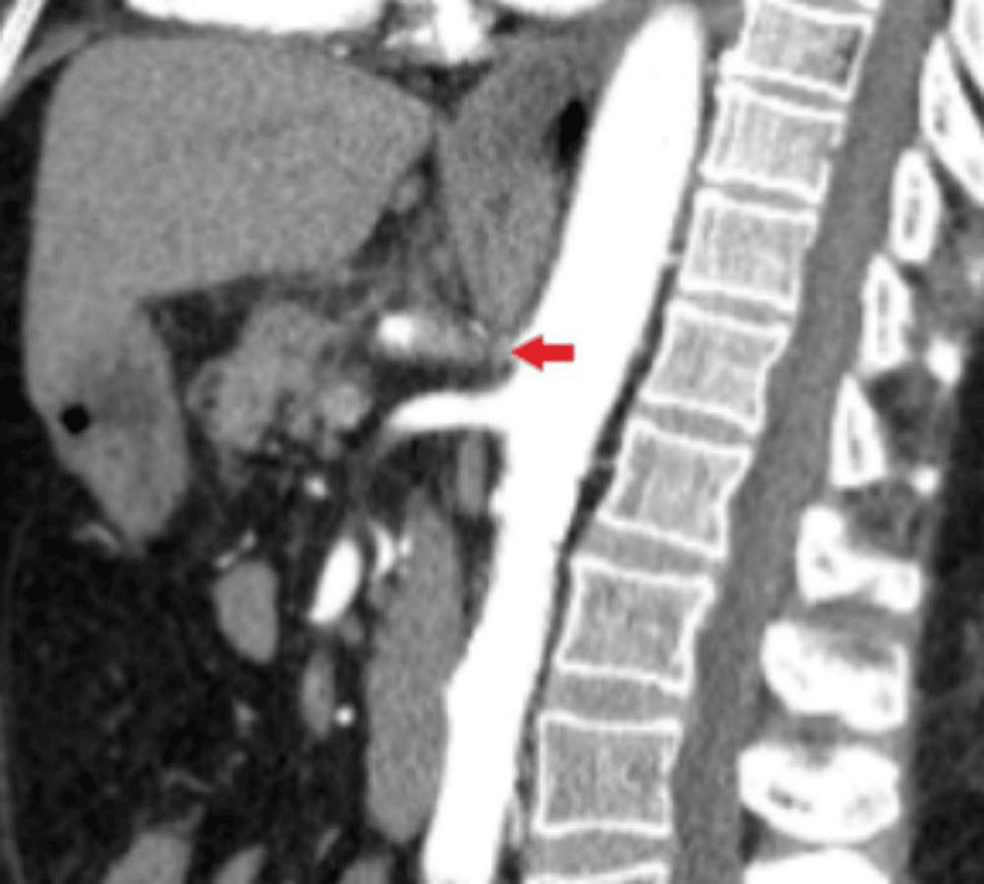
Computed tomography angiography (CTA) of the abdomen showing celiac artery thrombosis.

We describe a 65-year-old female with a past medical history of essential hypertension, hypothyroidism, generalized anxiety disorder, and hyperlipidemia who presented to the emergency department with a sudden onset of epigastric pain, associated with nausea that started a few hours after eating dinner. She described the pain as 'band-like', severe in intensity, with a burning sensation in the epigastric region. She denied any prior history of gallstones, pancreatitis, alcohol use, illicit drug use, hypertriglyceridemia, or any similar episodes. She also denied any recent abdominal trauma or fall. The home medications included losartan, levothyroxine, gabapentin, alprazolam, and topiramate. Initial evaluation in the emergency department showed a temperature of 97.7 degrees Fahrenheit, heart rate of 56 beats per minute, respiratory rate of 30 breaths per minute, blood pressure of 197/100 mmHg, and oxygen saturation of 100% on room air. Blood work showed a white blood cell count of 13.3 × 10^9^/L, hemoglobin of 13.3 g/dL, platelet count of 317 × 10^9^/L, blood urea nitrogen of 32 mg/dL, serum creatinine 1.29 mg/dL, aspartate aminotransferase (AST) of 31 units/L, alanine aminotransferase (ALT) of 38 units/L, alkaline phosphatase (ALP) of 119 units/L, total bilirubin of 0.5 mg/dL, calcium of 9.8 mg/dL, serum lipase of 429 U/L, and triglyceride of 171 mg/dL. Her coagulation profile showed a prothrombin time (PT) of 11.3 seconds, an international normalized ratio (INR) of 1, and activated partial thromboplastin time (aPTT) of 32 seconds. Computed tomography angiography (CTA) of the abdomen revealed inflammatory changes with fluid around the pancreas (Figure 1), thrombosis of the celiac artery, and patent gastro-hepatic trunk (Figure 2).

Abdominal ultrasound showed gallstones, which could be the underlying cause of acute pancreatitis in this patient. The patient was started on continuous infusion of intravenous heparin and vascular surgery was consulted to evaluate the patient. They recommended anticoagulation with apixaban rather than invasive intervention, as the patient was hemodynamically stable. The patient’s symptoms gradually improved and was discharged on the sixth day with apixaban for three months.

## Discussion

Vascular complications of acute pancreatitis are mostly associated with alcohol-induced pancreatitis, and necrotizing pancreatitis [9]. Portosplenomesenteric venous thrombosis (PSMVT), one of the common vascular complications, develops in 50% of patients with necrotizing acute pancreatitis but is rarely seen in the absence of necrosis [10]. Arterial thrombosis generally is less common and develops after the rupture of atheromatous plaque [11]. However, in acute pancreatitis, there are numerous changes in the microcirculation that can shift the homeostatic balance toward the formation of the clots, including elevated levels of D-dimers and fibrinogen, and activation of platelets [12]. The release of lipolytic and proteolytic enzymes into the bloodstream in the setting of acute pancreatitis leads to disruption of the vessel wall and activation of platelets and coagulation factors. Additionally, vascular stasis and vasospasm are induced by pressure necrosis, mass effect, and local inflammation generating a prothrombotic state [13].

Arterial complications in acute pancreatitis predominantly involve the splenic artery due to its location within the lesser sac in close proximity to the pancreas [14]. Among various arterial thromboses that have been reported, acute mesenteric arterial thrombosis is extremely morbid with a mortality rate reaching 90% in association with bowel infarction [15]. Specifically, celiac trunk thrombosis is extremely rare with only a few cases found in the literature. A case of pancreatitis-induced celiac artery thrombosis was reported in 2006, associated with partial hepatic and complete gastric necrosis. The patient underwent the Whipple procedure (pancreaticoduodenectomy), which was complicated by duodenal and esophageal stump leaks, managed conservatively initially, eventually requiring esophagojejunostomy [14]. Another patient with celiac trunk thrombosis due to pancreatic pseudocyst developed gastric and splenic necrosis and necessitated gastrectomy and splenectomy [16].

The clinical manifestations of pancreatitis-induced arterial thrombosis depend on the specific artery occluded. For example, splenic artery thrombosis can cause splenic infarction left gastric artery thrombosis can cause gastric infarction [14,16,17], but in our case, there was no evidence of any end organ infarction or necrosis. Vascular complications in pancreatitis are mostly associated with alcohol-induced pancreatitis [9]; however, in this case, the patient had developed gallstone-induced pancreatitis, diagnosed by noninvasive imaging measures. The treatment options for celiac artery thrombosis are not well documented; however, it is believed that splenic infarction can be managed medically but gastric infarction may require surgical intervention [17].

## Conclusions

We describe a case of celiac artery thrombosis in the setting of acute pancreatitis, in the absence of any necrotic changes, which was managed with anticoagulation therapy for three months. Arterial thrombosis in acute pancreatitis is associated with poor prognosis but there is no documented correlation between severity of pancreatitis and development of thrombosis. Therefore, it is vital to identify arterial thrombosis in the setting of acute pancreatitis to prevent ischemia but diagnosis may sometimes be difficult due to considerable peripancreatic edema that accompanies acute pancreatitis. However, providers must have a high index of suspicion in these situations.
